# Effect of UV_C_ and UV_A_ Photocatalytic Processes on Tetracycline Removal Using CuS-Coated Magnetic Activated Carbon Nanocomposite: A Comparative Study

**DOI:** 10.3390/ijerph182111163

**Published:** 2021-10-24

**Authors:** Negin Nasseh, Rasoul Khosravi, Narjes sadat Mazari Moghaddam, Shahabaldin Rezania

**Affiliations:** 1 Social Determinants of Health Research Center, Environmental Health Engineering Department, Faculty of Health, Birjand University of Medical Sciences, Birjand 9717853577, Iran; negin.nasseh2020@gmail.com (N.N.); khosravi.r@bums.ac.ir (R.K.); 2 Student Research Committee, Birjand University of Medical Sciences, Birjand 9717853577, Iran; 3 Department of Environment and Energy, Sejong University, Seoul 05006, Korea

**Keywords:** novel magnetic activated carbon nanocomposite, photocatalytic degradation, synthetic, pharmaceutical pollutant, tetracycline

## Abstract

In this study, we synthesized a novel MAC nanocomposite using almond’s green hull coated with CuS. The whole set of experiments have been conducted inside a batch (discontinuous reactor system) at room temperature. The effectiveness of different parameters in tetracycline removal pH (3, 5, 7, and 9), pollutant concentration (5–100 mg/L), nanocomposite dosage (0.025–1 g/L), and contact time (5–60 min) using newly synthesized nanocomposite were investigated. Based on the results, in the optimal conditions of pH = 9, nanocomposite dosage of 1 g/L, pollutant concentration of 20 mg/L, contact time of 60 min, and room temperature, 95% removal efficiency was obtained. In MAC/CuS/UV_C_ process, the removal of COD and TOC were 76.89% and 566.84% respectively meanwhile, these values in MAC/CuS/UV_A_ process were 74.19% and 62.11%, respectively. The results of nanocomposite stability and magnetic recovery illustrated that the removal efficiency was reduced by 1.5% in the presence of UV_C_ and 5% in the presence of UV_A_ lights during all six cycles. Therefore, this nanocomposite was highly capable of recycling and reuse. It can be concluded that considering the high potential of the synthesized nanocomposite, the photocatalytic efficiency of the MAC/CuS/UV_C_ process in tetracycline synthesis was higher than MAC/CuS/UV_A_ process.

## 1. Introduction

Among different pharmaceutical compounds, antibiotics seem to be the most attractive due to their potential for the development of antibacterial resistance on pathogenic bacteria [1]. The contamination of wastewaters with antibiotic compounds such as the pharmaceutical industry’s antibiotic compounds is considered a significant environmental challenge [2,3]. In addition, the increased consumption of antibiotics among human beings and livestock has made this compound to be persistent in aqueous resources. Tetracycline (TC) antibiotic is the second most prevalent antibiotic category in the world in terms of production and consumption. TC is one of the bacteriostatic antibiotics with a wide impact spectrum. These medicines are prescribed for the treatment of selective infections caused by Chlamydia (the main agent involved in trachoma, psittacosis, Salpingitis, Urethritis, and Lymphogranuloma venereum), Rickettsia (the main gent involved in Q fever) and mycoplasma [4].

Due to deficient and weak adsorption of TC antibiotics by organisms in human and animal bodies, the defecated TC would penetrate domestic sewage either through urination or feces and would finally contribute to environmental pollution [5,6]. Therefore, considering the extensive side-effects of this pollutant, the wastewaters containing antibiotic compounds must be treated by an effective method before being discharged into the environment. Active Oxidation Processes (AOPs) are considered as one of the most effective used methods for TC antibiotic removal. The AOP mechanisms are based on the production of free and activated radicals, especially, hydroxyl [OH^0^]. The non-selectivity property and high oxidation potential of these processes led to the more effective removal of organic and antibiotic compounds [7,8]. The photocatalytic synthesis process is a category of AOPs which has advantages such as cost-effectiveness, superior degradation, lack of secondary pollution, and low toxicity [9,10,11]. So far, multiple semiconductors have been used in photocatalytic synthesis as the nanocomposite for the photocatalytic synthesis of antibiotics. Some of them are tungsten trioxide [WO_3_] [12], cadmium sulfide [CdS] [13], zinc oxide [ZnO] [14], and titanium oxide [TiO_2_] [15]. 

In recent years, CuS has been extensively used for the removal of organic compounds from aqueous environments due to its high photocatalytic potential in the presence of UV as well as superior physical, chemical, and electromagnetic properties with a bandwidth of 2 EV [16]. These semiconductors are accompanied by various advantages including non-toxicity, applicability in room temperature, high resistance against chemical synthesis, high chemical stability in a wide pH range. Meanwhile, the most important advantages are the potential for adsorption of a wide spectrum of electromagnetic waves and high efficiency in photocatalytic degradation of organic compounds [17]. Despite the increased catalytic efficiency of these surfaces, the catalytic activity can be reduced after consecutive usages, because the reactive materials would be entrapped within these materials’ holes. Therefore, magnetic nanoparticles-based catalysts are considered as a good solution for the removal of organic pollutants as well as antibiotics. Additionally, it can be a good solution for recycling and application purposes [18]. 

Nowadays, activated carbon has effectively been used for the removal of pollutants from aqueous environments due to its high absorption potential, porous structure, and low costs involved. However, their large-scale application has been associated with problems such as filtration, scatteredness, turbidity development, and high costs of reduction [19]. Synthesis or combination of activated carbon with magnetic nanoparticles of (Fe_3_O_4_, MNPs) is considered as a good alternative for its optimal usage and production of wastewater with low turbidity [20]. These magnetic nanoparticles can finally be separate or remove from the aqueous environment using a magnet just after the reaction has been ended. Nowadays, agricultural by-products and plants can be used as the basis to produce activated carbon due to their abundance and low cost [21]. Almond is one of the indigenous plants of the eastern part of Iran and is considered the region’s commercial and economic product. Its green hull is discarded as waste and can be used for activated carbon production. Almond green hull alone has excellent biodegradability, but since photocatalytic processes require chemicals and semiconductors, this biodegradability will be reduced in the final composite [22]. There is a need to consider the negative impacts of antibiotic residuals on the environment and the updated nature of magnetic nanocomposites as well as the high efficiency of oxidation processes for the removal of organic pollutants. Therefore, the aim of this study was to evaluate the effect of UV_C_ and UV_A_ photocatalytic processes on TC removal using Magnetic Activated Carbon (MAC). The MAC has been derived from almond’s green hull and coated with CuS. The nanocomposite was characterized by FTIR, XRD, FESEM, DRS, VSM, and EDX and the effect of different variables such as pH, pollutant concentration, nanocomposite dosage, and contact time (5–200 min) has been studied.

## 2. Materials and Methods

### 2.1. Materials

The used materials in this study include TC hydrochloride salt [C_22_H_24_O_8_N_2_·HCL], purchased from Sigma Aldrich Company, Ethylene Glycol [C_2_H_6_O_2_], trivalent iron chloride [FeCl_3_·6H_2_O], bivalent iron chloride [FeCl_2_·4H_2_O], concentrated phosphoric acid, Hydrochloric acid [HCl], caustic soda [NaOH], sodium thiosulfate [Na_2_S_2_O_3_], Copper Sulfate [CuSO_4_], and 99% pure ethanol were purchased from Merk Company, Germany.

### 2.2. The Procedure of MAC Production

#### Magnetization of the Precursor

Initially, green almond hulls have been collected and then powdered. The resultant powder was sieved using 60 mesh size. Then, the resultant powder was sieved again using 200 mesh. Next, the obtained residual powder (with a size of 75–250 micron) was used for synthesizing MAC. For precursor magnetization, 6 g of the sieved powder were stirred along with 100 mL of sodium hydroxide on a 300-rpm shaker for 30 min. In the second stage, 200 mL of distilled water was heated to 60–70 °C on a 2-month balloon placed on a mixer and in the presence of nitrogen gas for 30 min at a temperature of 60–70 °C. In the third stage, 2 g of trivalent iron salt and 1 g of bivalent iron salt were added to the distilled water on the mixer. In the fourth stage, the resultant material from the first stage was added to the mentioned materials on the mixer. Finally, they were stirred for 1 h at 400 rpm speed at a temperature of 60 °C. without no injection of nitrogen gas. As the resultant material had alkaline pH, they were washed with distilled water and it has been dried in an 80 °C oven to reach the natural range [23].

### 2.3. Synthesis of Magnetic Activated Carbon (MAC)

In this stage, the magnetic and dried almond hulls were maintained in an isolated environment for 48 h and after being immersed in 10% phosphoric acid. Then, it was dried up in a vacuum oven for 3 h at 75 °C [24]. Next, the required material for furnace-treated carbonization has been prepared. To prevent oxygen penetration, the materials have been transferred into a lid cylindrical reactor. The steak reactor was transferred into the programmable furnace (HL40P controller model). Then, the furnace temperature raised to 550 °C with the speed of 300 rpm. Then, the materials were preserved in the same condition for 2 h [25]. Finally, after the furnace cooled down, the resultant carbon powder was immersed in the normal 3HCl for activation. Then, the activated materials were dispersed in an Ultrasonic device (Elmasonic E 30H model) with a frequency of 37 kHz for 1 h [26].

### 2.4. Sedimentation of Copper Sulfide on MAC

The 0.15 g of prepared MAC were dispersed within 20 mL of Ethylene Glycol (EG) inside the Ultrasonic device for 30 min. Next, the materials were transferred to a 500 mL volumetric flask inside an oil bath with a temperature of 120 °C. Then, 0.8 g of copper sulfate was added to the abovementioned suspension. Next, 1.9 g of Na_2_S_2_O_3_ was dissolved in 20 mL of EG, added to the suspension in the balloon, and stirred at reflux on a heater at 140 °C for 90 min. The obtained product was separated, washed once with ethanol and several times with deionized water, and finally dried in an oven at 80 °C for 5 h. After cooling down the volumetric flask, the resultant product was separated using an N_42_ magnet and several times with ethanol and once with the deionized water. Finally, it was dried up for 5 h at a temperature of 80 °C [27].

### 2.5. Characteristics of Synthesized Nanocomposite

To define the crystalline structure characteristics of MAC/CuS nanocomposite, X-ray Diffraction (XRD) analysis was administered using XRD instrument (X’Pert Pro model, Paralytical Co., Amsterdam, The Neterlands). Additionally, Field Emission Scanning Electron Microscopy (FESEM) technique (Sigma VP model, ZEISS Co., Germany, Berlin) was used to analyze the morphology, structure, average diameter, and estimation of the size of synthesized nanocomposite (MAC/CuS) in micro and nanoscales. The functional groups of the produced nanoparticles were identified using Fourier Transform Infrared (FT-IR) Spectroscopy (PerkinElmer Co., New York, NY, USA) within a wavelength range of 400–4000 cm^−1^. The magnetization of the synthesized magnetic nanoparticles was measured using Vibration Sampling Magnetometer (VSM, LBKFB model, Meghnatis Daghigh kavir Co., Iran, Tehran). The energy dispersive X-ray (EDX) Spectroscopy (Sigma VP-500 model, ZEISS Co., Germany) was used to measure the type and percentage of the constituent materials on the synthesized nanocomposite. Additionally, Diffuse Reflection Spectroscopy (DRS) Avapec-2048-TEC instrument with Avalamp DH-S setup was used to study the interaction between light and synthesized material as well as the photocatalytic properties of the nanocomposite.

### 2.6. Experiments for Photocatalytic Removal of TC

A TC stock solution was produced by considering its molecular weight as well as the purity percentage of the powder. This solution was synthesized weekly and preserved in a refrigerator at a temperature of 4 °C. The solution’s pH was adjusted using a pH meter (Knick-Calimatic model, Germany) by using NaOH and normal 0.1 hydrochloric (HCl) acid. All the above-mentioned experiments were conducted in 100 mL samples containing the pollutant and magnetic nanocomposite inside a 100 mL rector in the presence of 6 Watt UV_C_ and UV_A_ lamps in a batch. They were placed on a magnetic shaker with a mixture speed of 300 rpm. The effect of different variables including pH (3, 5, 7, and 9), pollutant concentration (5, 10, 20, 50, and 100 mg/L), nanocomposite dosage (0.025, 0.25, 0.5, and 1 g/L) and contact time (5–200 min) was studied. The samples were extracted from the reactor in different time intervals and the nanocomposite was separated using a magnet. Then, the remaining TC concentration was measured using a spectrophotometer device (UV/visible T80þ) at the wavelength of 358 nm [28].

The efficiency of the TC removal process was calculated according to the Equation (1).

(1) $$\mathrm{Removal}\;\%=\left(1-\frac{C_{t}}{C_{0}}\right)\times 100$$

where C_t_ and C_0_ are TCs (TC) concentrations in time t and its primary concentration (mg/L) respectively. R% displays the pollutant removal percentage.

### 2.7. Determination of Absorbent Surface (pH_zpc_)

At first, 0.01 M NaCl was prepared as the electrolyte (0.588 g/1000 cc of distilled water) and was poured into six Erlenmeyer flasks (100 mL each). The samples’ pH was adjusted in the range of 2–12 using NaOH and 0.1 M Hydrochloric Acid. About 0.2 g of MAC/CuS nanocomposite was poured into each Erlenmeyer and was shaken using a shaker with a 300-rpm speed for 24 h. Then, the final pH of the solutions was measured and the intersection of the two curves was determined as the isoelectric point’s pH by depicting pH_final_ curve in comparison with pH_initial_ curve [29].

### 2.8. Determination of Reaction Kinetics 

Analysis of catalytic processes for wastewater treatment, especially those wastewaters containing pharmaceutical compounds was performed through an analysis of reaction speed’s synthetic. According to the literature, the photocatalytic behavior of organic materials’ synthesis, especially the pharmaceuticals, follows a semi-first-order synthetic model, and the speed of heterogeneous catalytic reactions can be explained using Langmuir–Hinshelwood (L-H) synthetic model.

(2) $$R=k^{\prime }\theta =-\frac{\mathrm{dC}}{\mathrm{dt}}=k^{\prime }\left(\frac{\mathrm{KC}}{1+\mathrm{KC}}\right)$$

where r equals the speed of oxidation reaction (mgL^−1^ min^−1^), K′ is the constant of reaction’s speed (min^−1^), C is the pollutant’s concentration (mgL^−1^), K is the coefficient of reactor absorption (mg^−1^), and θ is the reactor’s site.

For solutions with a very low concentration (such as pharmaceuticals in water) with a K ≪1, the L-H equation (Equation (2)) can be simplified into a pseudo-first-order synthetic rule (Pseudo-First-order-kinetic (PFO^2^)):

(3) $$\frac{\mathrm{dC}}{\mathrm{dt}}=k_{\mathrm{obs}}C$$

(4) $$\ln\left(\frac{C}{C_{0}}\right)=-k_{\mathrm{obs}}t$$

where K_obs_ is the observed constant of pseudo-first-order kinetic speed (min^−1^), t is the reaction duration (min), C is the residual concentration after the intended time, and C_0_ is the initial pollutant concentration (mg/L).

### 2.9. Composite Recovery and Reuse Experiments

The stability and re-use potential of solid nanocomposite are among the significant parameters of this study. In this study, the experiments have been conducted in the obtained optimized conditions from photocatalytic experiments and within six different cycles to evaluate the possibility for further use of synthesized nanocomposite in the presence of UV_C_ and UV_A_ light for TC degradation. In each of these cycles, the intended magnetic nanocomposite was separated using an N_42_ magnet after each usage and washed several times using deionized water. Then, it dried up inside in an 80 °C oven. Finally, the residual concentration of the pollutant was separately measured after each cycle [30].

### 2.10. Conducting COD and TOC Experiments under Optimal Conditions

In this study, COD and TOC experiments were measured using ANA TOC (made in SGE Co., Australia) and Lovibond COD Vario Chekit Direct (made in Germany), respectively, based on 5310B and 5220D of the “Standard Manual for Water and Wastewater Experiments” [31].

## 3. Results

### 3.1. Characteristics of Synthesized Nanocomposite

#### 3.1.1. FESEM Analysis

**Figure 1:**
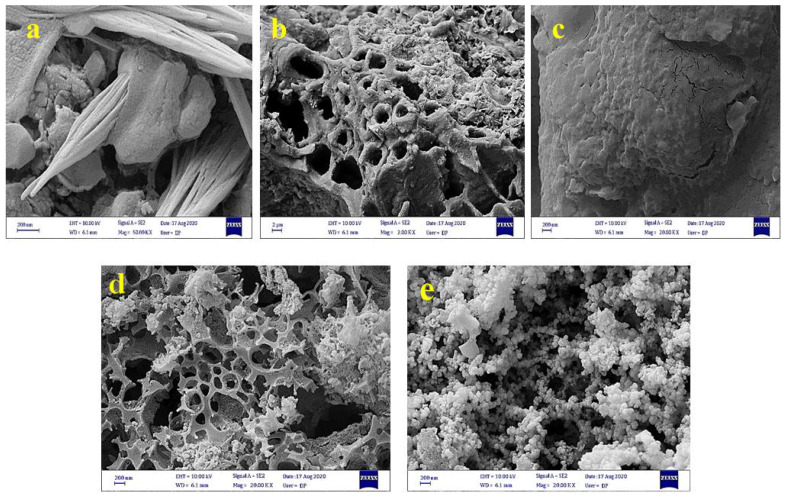
FESEM analysis. (**a**) almond’s green hull, (**b**) magnetic almond green hull (**c**) carbonized magnetic almond green hull, (**d**) magnetic carbon of almond’s green hull after activation, (**e**) magnetic carbon of almond’s green hull after activation loaded with CuS.

Figure 1 shows the FESEM images of the almond green hull, magnetic almond green hull, carbonated magnetic almond green hull, the activated carbon of magnetic almond green hull and the final nanocomposite coated with copper sulfide, respectively.

#### 3.1.2. XRD Analysis

**Figure 2:**
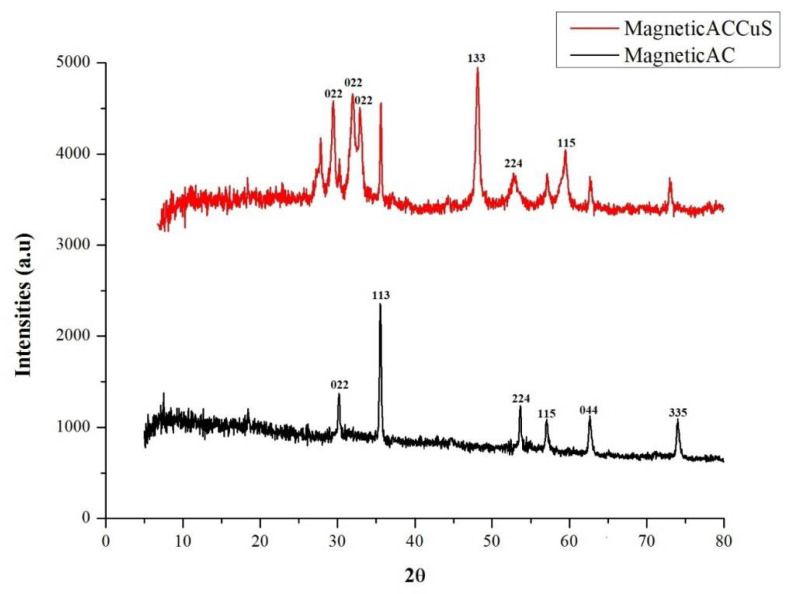
XRD analysis.

The X-ray Diffraction (XRD) pattern of MAC and MAC/CuS nanocomposite is shown in Figure 2. 

It should be noted that the crystal size of the magnetic nanocomposite synthesized in this study is also calculated from the “Scherrer” relation [32].

(5) $$D=\frac{0.98\lambda }{\beta \mathrm{COS}\theta }$$

where D is particles’ diameter, β is the peak’s width in half-height, θ is the diffraction angle in peak location, and λ is X-ray’s wavelength (λ = 0.1540 nm).

#### 3.1.3. DRS Analysis

**Figure 3:**
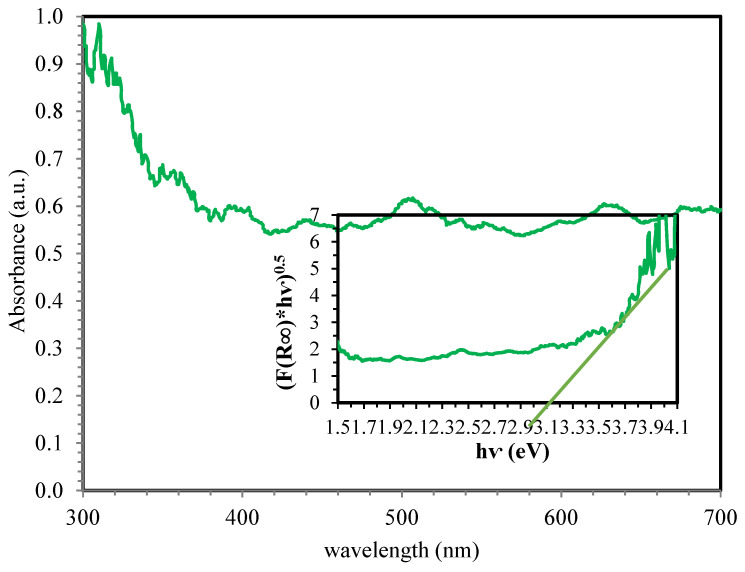
DRS analysis.

To investigate the effect of the materials used in the nanocomposite on the copper sulfide bond, DRS analysis was performed, the result of which is shown in Figure 3.

#### 3.1.4. EDX Analysis

**Figure 4:**
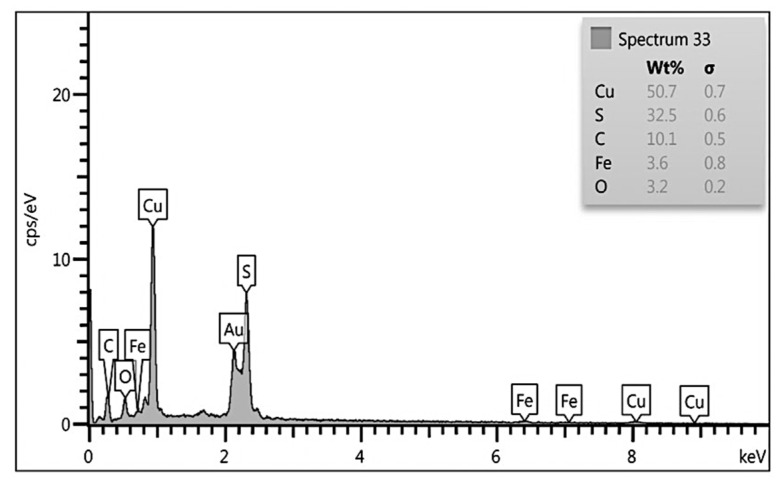
EDX analysis.

In order to investigate the elements present in the synthesized nanocomposite in this study, EDX analysis was performed. The results showed that the desired elements for the accuracy of nanocomposite synthesis, and are well defined in Figure 4.

#### 3.1.5. FTIR Analysis

**Figure 5:**
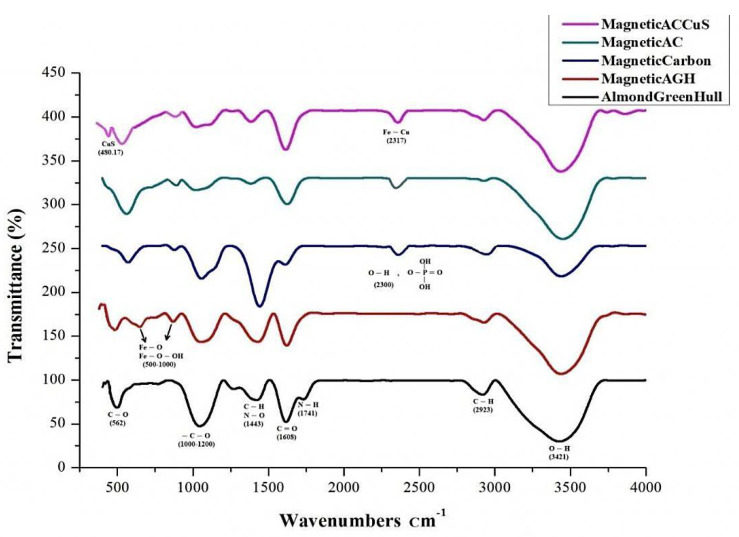
FTIR analysis.

The FTIR analysis of almond green hull, magnetic almond green hull, carbonated magnetic almond green hull, activated carbon of magnetic almond green hull and the final nanocomposite coated with copper sulfide, shown in Figure 5, respectively.

#### 3.1.6. VSM Analysis

**Figure 6:**
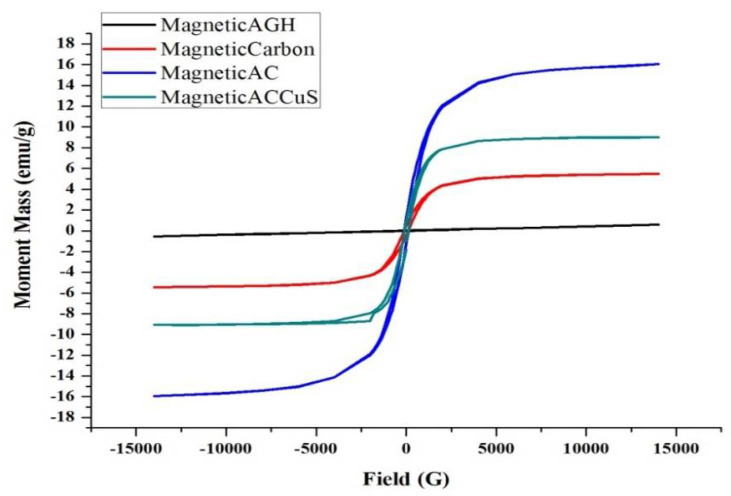
VSM analysis.

The results of VSM analysis for each of the compounds including magnetic almond green hull, magnetic carbon, magnetic activated carbon, and MAC/CuS are shown in Figure 6. 

### 3.2. Effective Parameters on TC Antibiotic Photocatalytic Synthesis

#### 3.2.1. Effect of pH

**Figure 7:**
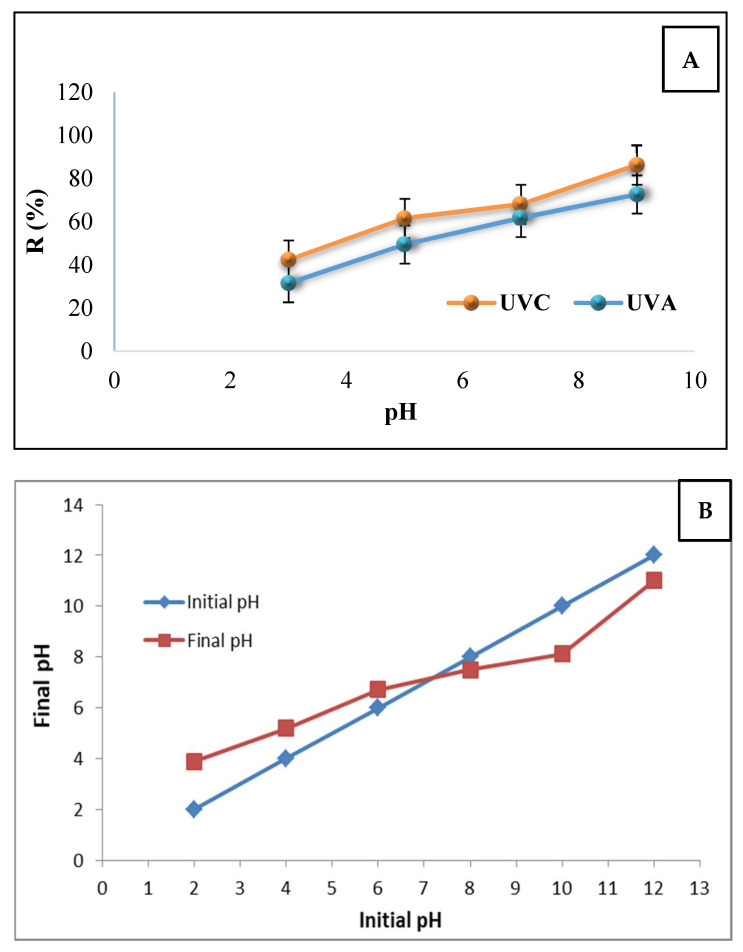
(**A**) Effect of pH in TC removal using MAC/CuS magnetic nanocomposite in the presence of UV_A_ and UV_C_ lights (TC concentration: 20 mg/L, nanocomposite dosage = 0.25 g/L, ambient temperature); (**B**) definition of MAC/CuS composite’s zeta potential.

Different studies have illustrated that pH is one of the significant and influential parameters in AOPs in the synthesis and removal of antibiotics [33]. It has a direct impact on other parameters such as pollutant decomposition rate, absorption valence, valence band’s oxidation potential, and the distribution of electrical load on nanocomposite’s surface [34]. In the present study, the impact of pH factor in TC photocatalytic synthesis can be observed in Figure 7A. Additionally, the surface load results of the synthesized nanocomposite are shown in Figure 7B. 

#### 3.2.2. Effect of Magnetic Nanocomposite Dosage

**Figure 8:**
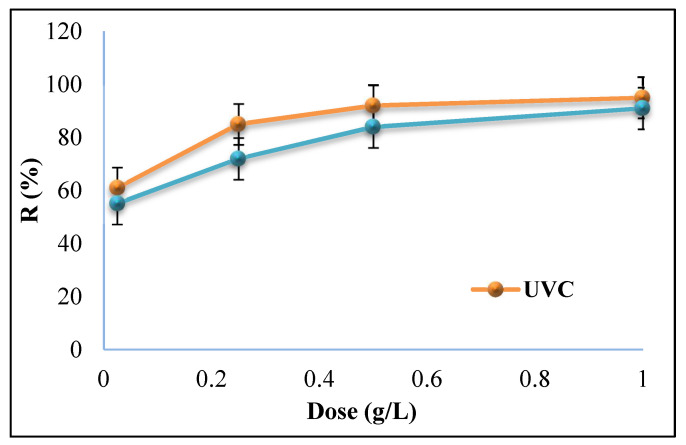
Effect of MAC/CuS nanocomposite dosage in the presence of UV_A_ and UV_C_ lights in TC removal (TC concentration of 20 mg/L, pH = 9, and ambient temperature).

Another effective factor in photocatalytic processes is magnetic synthesized nanocomposite dosage. In this study, different values (0.025, 0.25, 0.5, and 1 g/L) of the MAC/CuS nanocomposite have been studied to define the impact of nanocomposites’ optimal dosage on the photocatalytic removal of TC in the presence of UV_A_ and UV_C_ lights. All experiments were conducted under optimal conditions of (pH of 9 and TC concentration of 20 mg/L). As shown in Figure 8, the efficiency of TC removal was increased significantly by increasing the nanocomposite dosage to 0.5 g/L in the presence of UV_C_ and UV_A_ lights. On the other hand, from 0.5 to 1 g/L dosage of the nanocomposite, the removal efficiency had a slower rate. 

#### 3.2.3. Effect of TC Concentration and Contact Time

**Figure 9:**
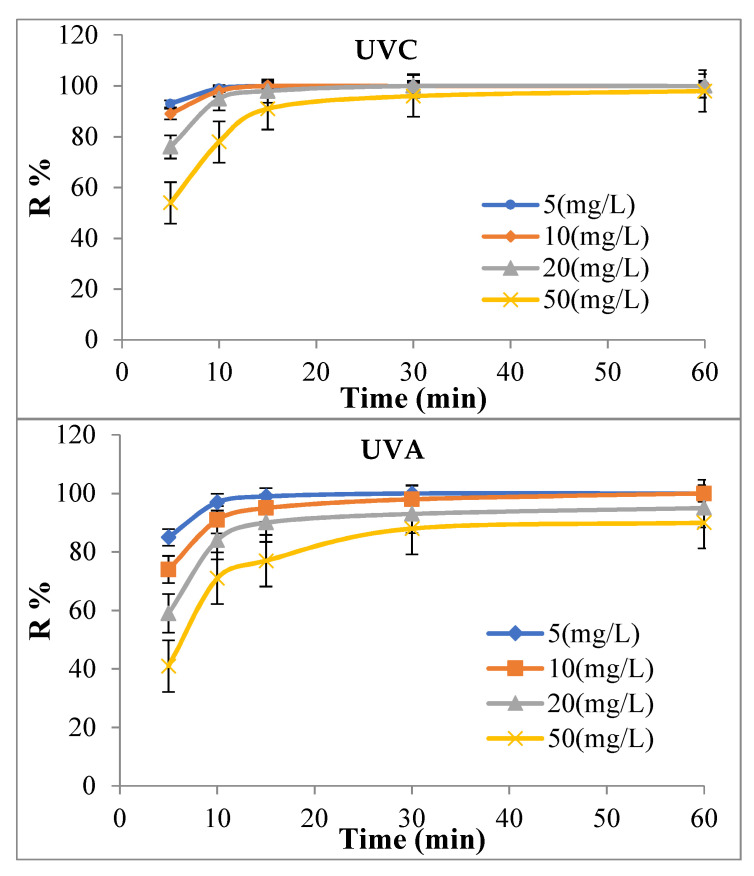
Effect of TC pollutant concentration variations on its removal using magnetic nanocomposite of MAC/CuS in the presence of UV_A_ and UV_C_ lights (nanocomposite dosage of 1 g/L, pH = 9, and ambient temperature).

To study the effect of TC concentration and contact time on the photocatalytic removal efficiency, different concentrations of TC (i.e., 5, 10, 20, and 50 mg/L) in different time intervals (i.e., 5, 10, 15, 30, and 60 min) was investigated. All the studies were performed under UV_A_ and UV_C_ lights in the optimal conditions (pH = 9 and MAC/CuS nanocomposite dosage of 1 g/L). The results of this section are shown in Figure 9.

### 3.3. Kinetics of Photocatalytic Decomposition of TC Antibiotic

**Figure 10:**
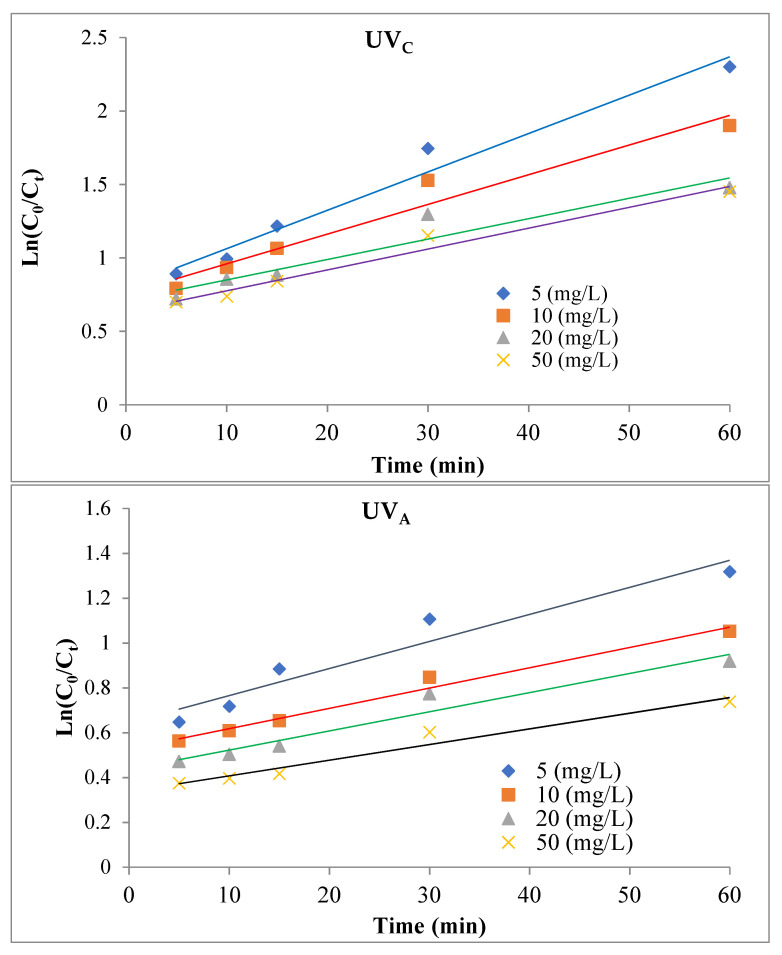
Kinetics of TC degradation reaction speed using MAC/CuS nanocomposite.

According to the literature, the synthetic of TC photocatalytic removal using MAC/CuS nanocomposite under UV_C_ and UV_A_ light and the destructive behavior of organic pollutants can be described through photocatalytic oxidation and pseudo-first-order synthetic model [35]. To this end, the experiments were conducted in optimal conditions (nanocomposite dosage of 1 g/L, pH = 9, t = 60 min, and different concentrations (5, 10, 20, and 50 mg/L). The results of the kinetics of the photocatalytic process in this study are shown in Figure 10 and Table 1.

### 3.4. Stability and Reusability of Magnetic MAC/CuS Nanocomposite in TC’s Photocatalytic Degradation Process

**Figure 11:**
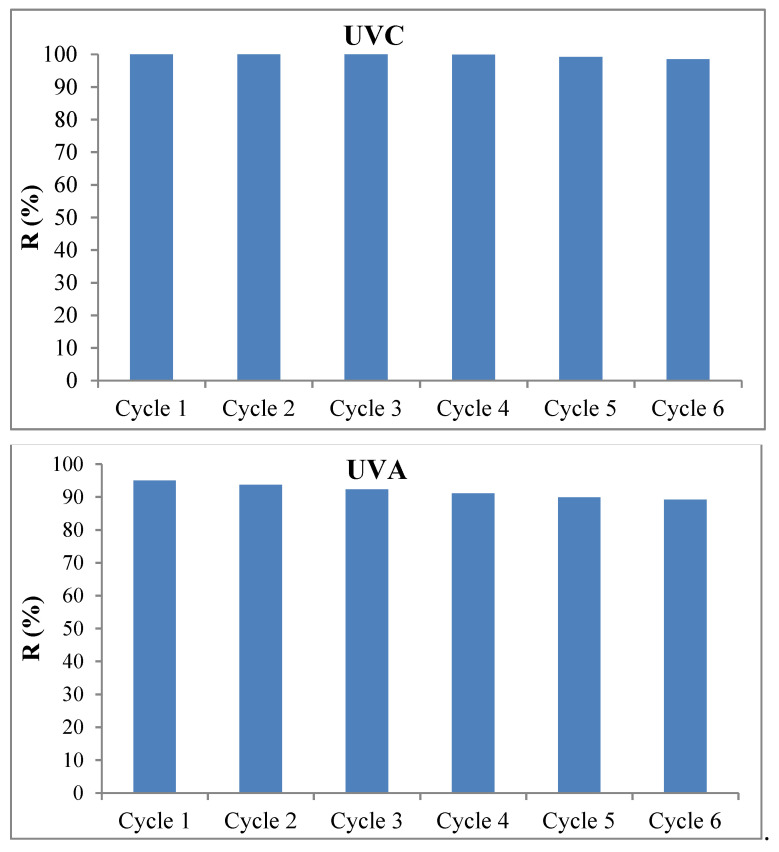
TC antibiotic degradation curve during MAC/CuS/UV_C_ and MAC/CuS/UV_A_ nanocomposite photocatalytic process throughout six stages of reuse (TC concentration = 20 mg/L; dosage = 1 g/L; t = 60 min, pH = 9).

Figure 11 displays the results of magnetic nanocomposite recovery in the photocatalytic process.

### 3.5. COD and TOC Removal Efficiency in the Optimal Conditions

**Figure 12:**
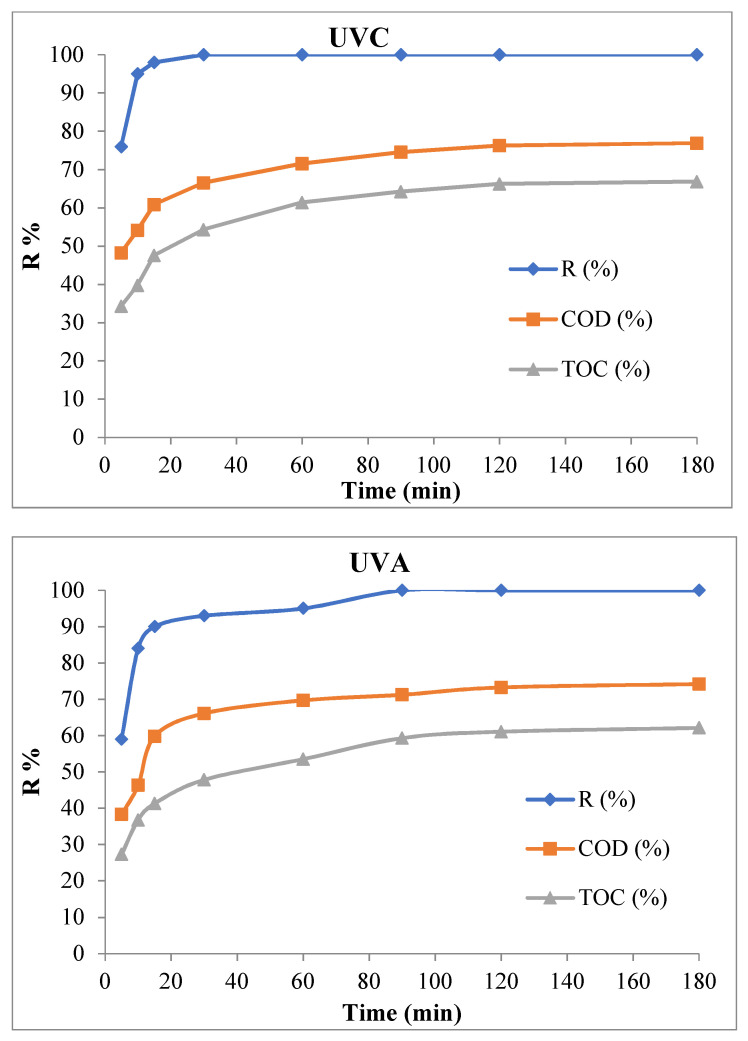
COD and TOC removal efficiency of synthetic TC pollutant under optimal conditions (pollutant concentration = 20 mg/L, nanocomposite dosage = 1 g/L, pH = 9, and ambient temperature).

COD and TOC removal efficiency of TC synthetic pollutants in a different time and optimal conditions (pollutant concentration = 20 mg/L, nanocomposite dosage = 1 g/L, pH = 9, ambient temperature) are illustrated in Figure 12.

### 3.6. TC Removal Mechanism throughout the Studied Process

The main reason for the application of semiconductor materials in photocatalytic reactors is the presence of two conduction and valence inside their structure. The area between the two bands is called the band gap. These reactions of degradation of TC in the photocatalytic process are as follows:

(6) $$\mathrm{CuS}\overset{h\vartheta }{\to }\;\mathrm{CuS}\;\left(e^{-}+{\;h}^{+}\right)$$

(7) $$\left(h^{+}\right)+H_{2}O\to \;{}^{•}\text{OH}+{\text{H}}^{+}$$

(8) $$\left(h^{+}\right)+{\mathrm{OH}}^{-}\to \;{}^{•}\text{OH}$$

(9) $$e^{-}+{\;O}_{2}\to \;{}^{•}O_{2}^{-}$$

(10) $${}^{•}O_{2}^{-}+{\;H}^{+}\to \;{}^{•}{\text{HO}}_{2}$$

(11) $$2\;{}^{•}{\text{HO}}_{2}\to \;H_{2}O_{2}+O_{2}$$

(12) $$H_{2}O_{2}+\;{}^{•}{\text{O}}_{2}^{-}\to \;{\text{OH}}^{-}+{}^{•}\text{OH}+O_{2}$$

(13) $$H_{2}O_{2}+{\;e}^{-}\to {\mathrm{OH}}^{-}+\;{}^{•}\text{OH}$$

(14) $$H_{2}O_{2}+\left(h^{+}\right)\to 2\;{}^{•}\text{OH}$$

Finally, TC pollutants would be destructed through valence-band hole through direct oxidation or would be destructed through hydroxyl radicals and an indirect oxidation process.

(15) $$TC+({}^{•}\text{OH},{}^{•}{\text{HO}}_{2},O_{2}^{-}\;\text{or}\;(h^{+})\to Intermediates\to final\;products$$

**Figure 13:**
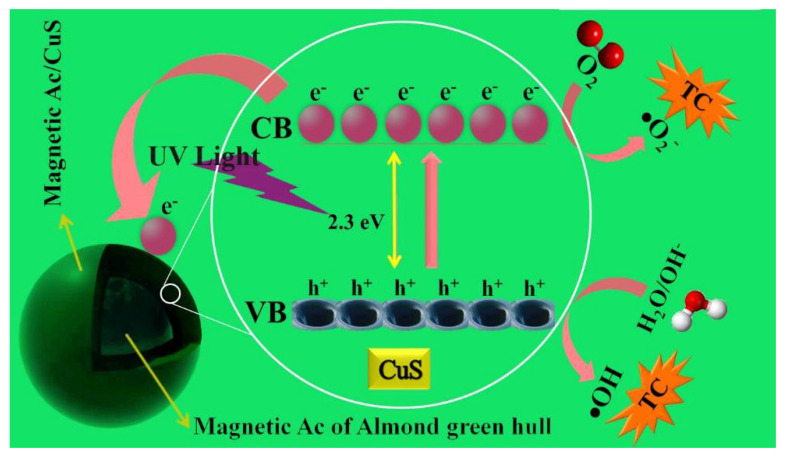
The photocatalytic removal mechanism of tetracycline.

Figure 13 depicts the mechanism of the photocatalytic removal process of tetracycline using the nanocomposite synthesized and the reactions taking place in this process.

## 4. Discussion

### 4.1. Characteristics of Synthesized Nanocomposite

#### 4.1.1. FESEM Analysis

Using the field emission scanning electron microscope, the shape, average diameter, and surface details of the nanoparticles and nanocomposites in this study were investigated. (Figure 1a) show FESEM images of the almond’s green hull. As can be seen, some holes and gaps appeared on the absorbent’s surface. In addition, the magnetic almond hull structure had a uniform and favorable placement of iron particles in the almond’s green hull (Figure 1b). Meanwhile, the carbonized magnetic almond green hull had minimum holes which were evidence of carbon precursors on the surface (Figure 1c) [36]. Figure 1d displays a micrograph with a magnification ratio of 2000 KX taken from magnetic carbon of almond’s green hull after activation. Based on this, numerous irregular holes were developed on the surface of the almond hull’s magnetic carbon, following activation procedure. Besides, ultrasonic waves in aqueous environments lead to cavitation and development of free radicals and these free radicals and consequently resulted in the oxidation of organic compounds [37]. Figure 1e shows the final form of MAC/CuS after loading CuS on the activated carbon which derived from magnetic almond’s green hull. 

#### 4.1.2. XRD Analysis

X-ray diffraction (XRD) spectroscopy is a rapid analytical technique used to detect the type of material as well as its phase and crystalline properties. Different materials have different diffraction patterns due to different atomic arrangements. So, the diffraction pattern of each combination is unique. The peaks related to the presence of iron in MAC were observed in 2θ which equal 30.78, 35.46, 53.84, 57.52, 63.02, and 74.53. Considering the ICSD 159,976 reference card, these crystalline sheets were for the spinel structure of the Fe_3_O_4_ inverse cube.

As can be seen, the CuS peaks were observed in 29.35, 31.85, 32.13, 48.04, 51.0, and 59.89 (ICSD 158743). The size of CuS nanoparticles loaded on MAC was 29 nm as calculated by Debye-Sherrers equation by considering the Full-Width Half Maximum (FWHM) of the most intense diffraction peak. Although, In the FESEM analysis, it was found that the obtained nanoparticles tended to accumulate because of their magnetic properties and the particle size was 64 nm which was bigger than the calculated size from Shrer equation. This can be justified in the light of the assertion that XRD analysis that was mainly concerned with the crystal size; however, FESEM analysis points to the particle size and it is noteworthy that any particle was composed of a group of crystals.

#### 4.1.3. DRS Analysis

According to the literature, CuS has 2 bandgaps; however, based on the results of this analysis and the drawing of the Tauc diagram according to Equation (αhυ)^n^ = k(hυ-Eg) (Where α = absorption coefficient, h = plank constant, ν = frequency, Eg = band gap, k = constant, n = for Indirect energy gap = ½, for direct energy gap = 2), to estimate the energy gap of the copper sulfide semiconductor optical band in the synthesized MAC/CuS nanocomposite structure, it was determined that the presence of other materials in the synthesized MAC/CuS slightly changed the band gap of copper sulfide (band gap = 3.1).

#### 4.1.4. EDX Analysis

In EDX analysis, by measuring the wavelength (or energy) and the number of x-rays emitted per second, the elements present in the sample or performing qualitative analysis as well as the amount of element present in the sample or performing quantitative analysis can be detected. The results of the EDX analysis of the nanocomposite are provided in Figure 4, which clearly shows the presence of C, O in this substance which is mostly related to the almond green hull. In addition, this figure also shows the presence of iron on the hull in a 1 to 3 ratio. Finally, the results of the EDX analysis of the finally synthesized composite clearly show the bold presence of elements related to the semiconductor copper sulfide that makes up the top layer of the composite. Additionally, as shown, the elements of copper, sulfur, and carbon are the highest weight percent of the synthesized nanocomposite, respectively, and oxygen has the lowest percentage.

#### 4.1.5. FTIR Analysis

This type of spectroscopy can identify different functional groups on molecular compounds and thus show the possible structure of the compounds. The FTIR analysis of almond’s green hull is shown in Figure 5. As can be seen, the –C–O peak appeared in 1000–1500 cm^−1^ which can be observed in all FTIR for the almond green hull, magnetic almond green hull, magnetic carbon, magnetic activated carbon, magnetic activated carbon, and magnetic-activated carbon coated with CuS. The peak in wavelengths of 1443 cm^−1^ was attributed to the functional groups of C-H or N-O while the peak in wavelength of 1608 cm^−1^ corresponded to C=O factorial group. It should be noted that the peak for C-H and O-H factorial groups appeared in the wavelength of 3421 cm^−1^. In addition, the short peak with a wavelength of 1741 cm^−1^ in the respective spectrum for the almond green hull was the representative of N-H factorial group [38].

Since iron was used in all spectra for the magnetic almond green hull, magnetic carbon, and magnetic activated carbon, thus, the peaks in 500–1000 cm^−1^ can be attributed to Fe-O-OH and Fe-O factorial groups [39]. In addition, within the two spectra for almond’s green hull and magnetic almond green hull in the wavelength of 562 cm^−1^, the C-O factorial group appeared [40]. 

Based on Figure 5, the peak for the C-O factorial group was diminished due to the carbonization of the magnetic almond green hull and the iron’s peak becomes more visible. Moreover, since phosphoric acid was used for carbonization of the magnetic almond green hull, the two spectra for magnetic carbon and activated magnetic carbon within the wavelength of 2300 cm^−1^ are related to O-H and phosphate factorial groups were highly visible. In addition, the absorption band existing in 2317 cm^−1^ in MAC/CuS nanocomposite spectrum was related to the Fe-Cu bond’s vibration that overlapped with the peak for O-H and phosphate factorial groups. Finally, the absorption band within 480.17 cm^−1^ was related to the metal bond’s vibrations with sulfur’s heteroatom, which correctly justifies the CuS bond [41].

#### 4.1.6. VSM Analysis

In this study, the magnetism of nanoparticles and nanocomposites synthesized by analysis (VSM) was investigated and measured. The value of magnetic saturation of each of these compounds was 0.5, 5.1, 16.8, and 9.2, respectively. Magnetic almond green hull did not possess any magnetic power in the presence of iron. When it was placed on an N_42_ magnet, there was no magnetic reaction which proved by VSM analysis. This can be due to the different contents of the almond green hull. Through carbonization of the magnetic green almond hull in the furnace and the subsequent loss of many compounds, the magnetic property of the iron was observed. This magnetic potential was evidence of its magnetic curve. However, due to maintaining many of the organic compounds on the nanocomposite’s surface in the form of bitumen-like material, the magnetic potential of the carbon was reduced. Then, the activation procedure can remove these bitumen-like materials from the nanocomposite’s surface to enhance its magnetic potential significantly. Therefore, by considering the VSM analysis results, the carbon obtained from magnetic almonds green hull had the highest magnetic potential. As loading CuS on the derived activated carbon was partially reduced the magnetic power.

### 4.2. Effective Parameters on TC Antibiotic Photocatalytic Synthesis

#### 4.2.1. Effect of pH

The alkaline environment has facilitated the optimal conditions for the removal of TC antibiotics. The scholars have argued that TC is an amphoteric molecule that is composed of various factorial groups (e.g., dimethylamino group, phenolic diketone group, tricarbonyl, and methane group) [42]. The acidic constant (pK_a_) of each factorial group in aqueous environments with variable pH is different. This variation can be protonated in the acidic range of pH <4, and become neutral in acidic to a neutral range (4 <pH>7.5) and in the form of anion in the neutral to alkaline range (7.5 <pH>10) [43]. Therefore, the dominance of one or multiple species of TC within the environment defines the impact of pH variations on the synthesis speed and quantum efficiency of TC [44]. Different studies declared that in case the tricarbonyl methane factorial group would be the environment’s most dominant factor, TC synthesis would be the lowest. Moreover, the maximum TC removal percentage is observed when dimethyl amino is the most dominant form, such that the electron density of the TC group is higher in alkaline environments compared with acidic environments [45,46]. Besides, the neutral and alkaline pH ranges have the maximum oxidation valence for TC pollutants within photocatalytic processes. The present study confirmed the same results for a pH of 3 as the pollutant removal percentage in a time interval of 10 min reached 42.3% and 31.5% in the presence of UV_C_ and UV_A_ lights, respectively. Hence, the removal of TC increased to 68.12% in pH of 7 and 86.29% in the pH of 9 in the presence of UV_C_ light. The results showed that the TC removal was 61.8% in pH of 7 and 72.7% in pH of 9 in the presence of UV_A_ light [46,47]). In separate studies and during different photocatalytic processes, it was reported that with increasing pH, the removal percentage of TC increases significantly, which is consistent with the results of this study.

To predict the behavior of the synthesized nanocomposite, the definition of isoelectric point (pH_ZPC_) is significant. As mentioned before, many studies have pointed out that the TC molecule in acidic pH is protonated (H_4_TC^+^) and its dominant loading is positive [48]. Besides, considering the study results, the surface of the magnetic nanocomposite was within a positive acidic pH range [44]. Due to the developed desorption force in acidic pH, the TC absorption on the surface of the MAC/CuS magnetic nanocomposite can be reduced [29]. Based on the results, the isoelectric point of the studied magnetic nanocomposite was equal to 7 (Figure 7B)

In addition, in acidic pH values, the propensity of the nanocomposite particles for agglomeration reduced the removal efficiency of the process as well as the access level of nanocomposite for the absorption of TC within aqueous solutions. In the neutral and alkaline pH, OH radicals are the most significant oxidation agents which are developed as a result of the interaction between hydroxide (OH^−^) ions and positive holes [49]. However, the most significant oxidation species in acidic pH are positive holes [48].

#### 4.2.2. Effect of Magnetic Nanocomposite Dosage

The results showed that the pollutant removal efficiency in 0.5 and 1 g/L dosages was 92% and 95% in the presence of UV_C_ light, and 84% and 91% in the presence of UV_A_ light, respectively. As the nanocomposite dosage increased, the nanocomposite was declined toward agglomeration and sedimentation due to the increase in magnetization potential [50]. This can be explained by the reduction in accessible active sites for photon absorption on its surface which consequently leads to the reduction in pollutant removal efficiency [51]. In addition, it can be predicted that increasing nanocomposite dosage to higher than 1 g/L may develop turbidity and disturb the process of light transmission inside the solution. Thus, the optimal nanocomposite dosage for MAC/CuS/UV_C_, and MAC/CuS/UV_A_ was 1 g/L.

#### 4.2.3. Effect of TC Concentration

The results in Figure 9 show that the removal efficiency decreased by increasing TC concentration at the interval of 10 min and a concentration of 5 mg/L In addition, TC removal reached in the presence of UV_C_ 99% and 97% in the presence of UV_A_. However, the TC removal in concentrations of 10, 20, and 50 mg/L in the presence of UV_C_ light was 98%, 95%, and 78%, respectively. From these findings, it can be stated that the higher the concentration of the pollutant, the more light is scattered and cannot reach the entire surface of the nanocomposite particles to activate the photocatalytic process. Therefore, all nanocomposite parts stayed unaffected by UV_C_ and UV_A_ lights. Although, the synthesis value reduced significantly. In addition, by increasing in TC concentration, its absorption rate on MAC/CuS nanocomposite was increased and the nanocomposite efficiency was reduced. It was due to the increased reaction between TC pollutants, OH radicals, and electron holes in low concentrations. The results of Nasseh et al. (2018) showed that the photocatalytic removal of TC using magnetic FeNi_3_/DiO_2_/CuS nanocomposite was similar to the obtained results of this study [16]. It is noteworthy that know that the radiation of UV_C_ and UV_A_ lights on their own did not significantly affect the TC removal efficiency. The TC removal efficiency in the presence of UV_C_ light was less than 40% and in the presence of UV_A_ light was less than 30% after 60 min [52].

### 4.3. Kinetics of Photocatalytic Decomposition of TC Antibiotic

Based on the results in Table 1 and Figure 10, it can be concluded that the increase in pollutants’ concentration was negatively correlated with the reaction speed constant (K_obs_). In this study, the obtained R^2^ determination coefficient was near 1 which showed that the pseudo-first-order synthetic model was the best model for describing reaction’s speed in this process. It should be noted that the increase of intermediate products’ concentration to high concentrations and limitation of active hydroxyl radicals, can reduce the decomposition speed constant [1].

### 4.4. Stability and Reusability of Magnetic MAC/CuS Nanocomposite in TC’s Photocatalytic Degradation Process

The results showed that TC degradation after six cycles was 98.45% and 89.17% in the condition of UV_C_ and UV_A_ lights, respectively (Figure 11). The photocatalytic efficiency was reduced for 1.15% and 5% in the presence of UV_C_ and UV_A_ lights, respectively. This can be due to the variation of nanocomposite structure under the impact of optical reactions as well as decreased amount of nanocomposite (5% of the nanocomposite cannot be separated using a magnetic field) [53]. The recovery of nanoparticles through various methods including centrifuge, and filtration would be costly and time-consuming. On the other hand, the easy separation of synthesized nanocomposite from the solution is considered a favorable feature for nanocomposite recovery. Magnetic separation also has a lower mass loss than other conventional separation methods, which can have a significant impact on the recovery cost.

### 4.5. COD and TOC Removal Efficiency in the Optimal Conditions

Based on the results the TC removal efficiency and COD and TOC in MAC/CuS/UV_C_ process in 180 min were 100%, 76.89%, and 66.84% respectively. Besides, the reported values for the same parameters in MAC/CuS/UV_A_ process were 100%, 74.19%, and 62.11%, respectively (Figure 12). It was due to the incomplete antibiotic decomposition throughout the photocatalytic process. In other words, while a major portion of the antibiotic completely breaks down into the expected mineral compounds, i.e., H_2_O and CO_2_, the rest of it turns into organic by-products. These organic intermediate products confirm the TOC and COD removal efficiency in comparison with the removal of TC antibiotics [54].

### 4.6. TC Removal Mechanism throughout the Studied Process

The photocatalytic process of this study progresses as follows. First, the nanocomposite particle absorbs a photon with energy equal to or greater than its bandgap, which results in the creation of an electron-hole pair following the excitation of the electron from the valence band to the conduction band (reaction 6). The high oxidation potential of the holes contributed to the formation of hydroxyl radicals throughout oxidation and reduction reactions in the presence of particles such as OH^−^, and oxygen which are absorbed on the nanocomposite’s surface (reactions 7 and 8). On the other hand, photon-produced electrons can produce superoxide (O_2_^−^) and other activated oxygen species such as hydrogen peroxide as they were trapped in dissolved oxygen (reactions 9–11). Moreover, more free radicals are produced as a result of hydrogen–peroxide-initiated reactions [55,56].

Generally, the main five reaction stages involved in the heterogeneous photocatalytic process are (1) the penetration of reactor materials on the nanocomposite’s surface; (2) the absorption of reactors on the surface; (3) reaction on nanocomposite surface; (4) desorption of the reaction products from the surface; and (5) separation of the products from the surface and their penetration inside the solution (Figure 13).

## 5. Conclusions

In this study, we compared the UV_A_ and UV_C_ photocatalytic processes for TC removal using MAC obtained from the almond green hull, coated with CuS. Additionally, the effects of different variables including pH, nanocomposite dosage, and pollutant concentration throughout different contact times were investigated. The results showed that the new nanocomposite can be applied as a recoverable and efficient nanocomposite for TC degradation. In addition, MAC/CuS/UV_C_ photocatalytic process was more efficient in TC antibiotic degradation compared with MAC/CuS/UV_A_ process in optimal conditions (pH = 9, nanocomposite dosage = 1 g/L; pollutant concentration = 20 mg/L; contact time = 60 min; ambient temperature). The TC degradation for MAC/CuS/UV_C_ was 100%, while it was 95% by MAC/CuS/UV_A_ photocatalytic process. In addition, the degradation speed synthetic for both MAC/CuS/UV_C_ and MAC/CuS/UV_A_ processes followed the pseudo-first-order model. The results of nanocomposite stability and magnet recovery suggested that the TC degradation was 98.45% in UV_A_ and 89% in UV_C_ process after six usages of the photocatalyst. Therefore, considering the relatively simple synthesis and high efficiency of the synthesized nanocomposite throughout the TC photocatalytic degradation process, it can be considered as a favorable nanocomposite in either removal or degradation of pharmaceutically resistant organic pollutants.

## Figures and Tables

**Table 1:** Pseudo-first-order synthetic parameters for TC decomposition.

UV_C_			
Concentration (mg/L)	Equation	K_0_ (min^−1^)	R^2^
5	Y = 0.025x + 1.084	25 × 10^−3^	0.9824
10	Y = 0.0181x + 1.0046	18.1 × 10^−3^	0.9715
20	Y = 0.0138x + 0.8292	13.8 × 10^−3^	0.9895
50	Y = 0.0116x + 0.778	11.6 × 10^−3^	0.9631
t_1/2_ = 0.693/K_0_			
**UV_A_**			
Concentration (mg/L)	Equation	K_0_ (min^−1^)	R^2^
5	Y = 0.0257x + 1.0758	25.7 × 10^−3^	0.9933
10	Y = 0.0157x + 0.9309	15.7 × 10^−3^	0.9938
20	Y = 0.0106x + 0.9305	10.6 × 10^−3^	0.9849
50	Y = 0.0096x + 0.8483	9.6 × 10^−3^	0.9849
t_1/2_ = 0.693/K_0_			

## Data Availability

Not applicable.
